# Unraveling the Abnormal Molecular Mechanism of Suicide Inhibition of Cytochrome P450 3A4

**DOI:** 10.1021/acs.jcim.2c01035

**Published:** 2022-12-02

**Authors:** Yang Zhou, Junhao Li, Glib Baryshnikov, Yaoquan Tu

**Affiliations:** † School of Pharmacy, Jinan University, 601 Huangpu Avenue West, Guangzhou510632, China; ‡ Department of Theoretical Chemistry and Biology, KTH Royal Institute of Technology, 114 28Stockholm, Sweden; § Laboratory of Organic Electronics, Department of Science and Technology, Linköping University, 60174Norrköping, Sweden

## Abstract

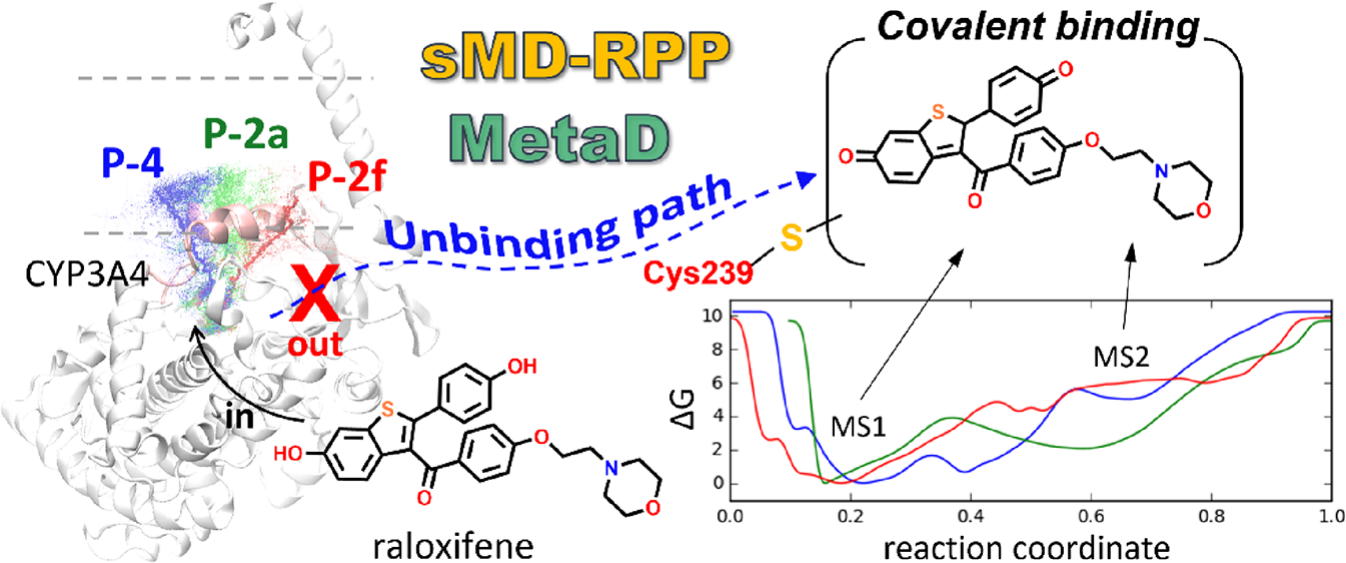

Suicide inhibition of the CYP3A4 enzyme by a drug inactivates the enzyme in the drug biotransformation process and often shows safety concerns about the drug. Despite extensive experimental studies, the abnormal molecular mechanism of a suicide inhibitor that forms a covalent bond with the residue far away from the catalytically active center of CYP3A4 inactivating the enzyme remains elusive. Here, the authors used molecular simulation approaches to study in detail how diquinone methide (DQR), the metabolite product of raloxifene, unbinds from CYP3A4 and inactivates the enzyme at the atomistic level. The results clearly indicate that in one of the intermediate states formed in its unbinding process, DQR covalently binds to Cys239, a residue far away from the catalytically active center of CYP3A4, and hinders the substrate from entering or leaving the enzyme. This work therefore provides an unprecedented way of clarifying the abnormal mechanism of suicide inhibition of the CYP3A4 enzyme.

## Introduction

Cytochrome P450 (CYP) enzymes are essential for the biotransformation of a broad range of structurally diverse molecules including drugs, chemical carcinogens, steroids, and fatty acids.^1,2^ CYP3A4 is one of the most important drug-metabolizing isoforms of CYP enzymes since it interacts with more than 50% of clinically used drugs.^1^ The activity of CYP3A4 can be inhibited reversibly or irreversibly by xenobiotics, which will most likely further influence the clearance of toxins and the body’s response to the co-administered drugs. Irreversible CYP3A4 inhibition, also called suicide inhibition of CYP3A4, is caused by the covalent binding of the reactive metabolites or intermediates, called suicide inhibitors, to the enzyme in a biotransformation process. Suicide inhibition of CYP3A4 has been believed to be detrimental to drug biotransformation, and drugs on the market could be withdrawn because of the safety risks related to suicide inhibition.

**Figure 1:**
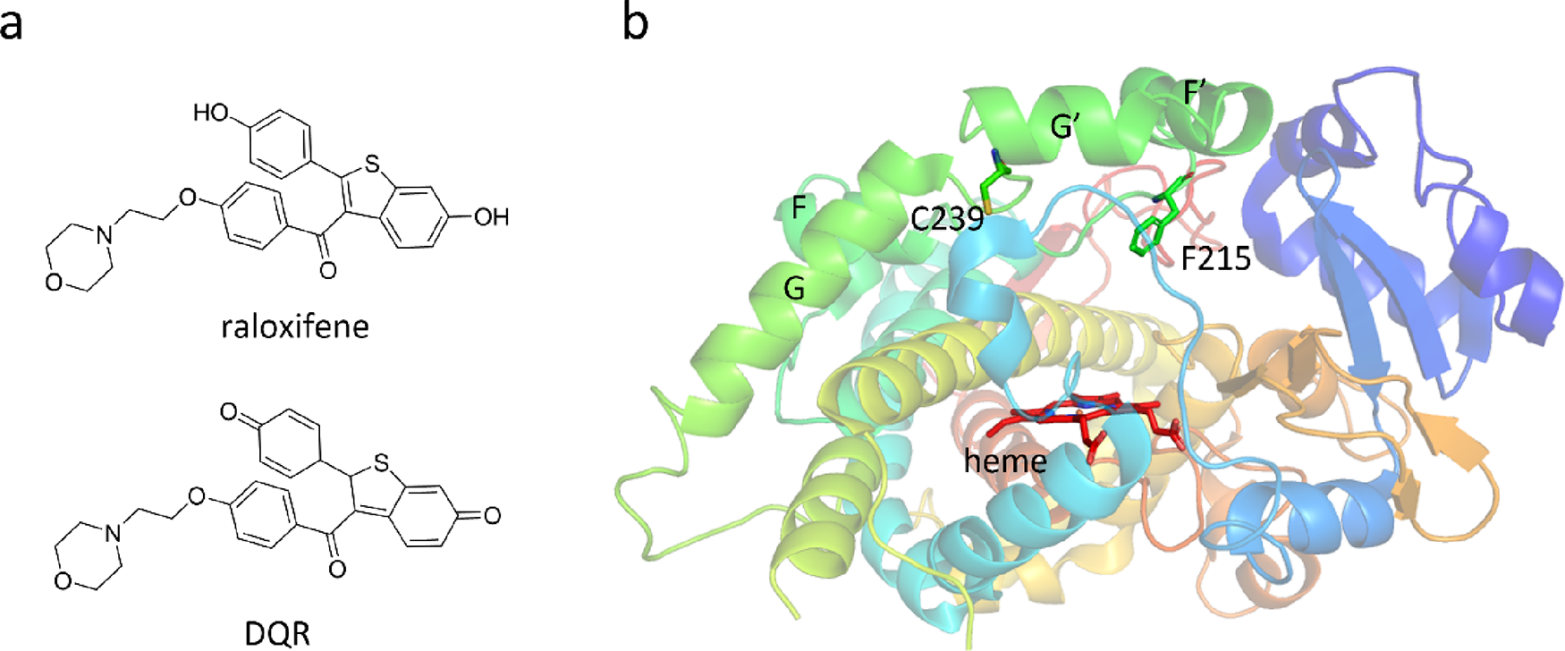
(a) Chemical structures of raloxifene and DQR. (b) Crystal structure of CYP3A4 (PDB: 1TNQ).

Raloxifene is a selective estrogen receptor modulator used for the treatment of osteoporosis in post-menopausal women.^3^ Recent studies demonstrate that raloxifene is a special suicide inhibitor of CYP3A4.^4−7^ Usually, a suicide inhibitor covalently binds to heme or the residues of the catalytically active pocket of a CYP enzyme. However, analysis shows that this is not the case for raloxifene. In vitro studies indicate that CYP3A4 converts raloxifene to produce several species including diquinone methide (DQR, Figure 1a).^5,6^ DQR covalently binds to the enzyme and inhibits its activity. A mass spectral analysis shows that there exists a single covalent bond between DQR and CYP3A4.^8^ Further analyses of peptides following digestion with proteinase K reveal that this covalent bond is localized on residue Cys239^5,6^ (Figure 1b). Moore et al. showed that residue Phe215 plays an important role in the dehydrogenation and hydroxylation selectivity of raloxifene.^9^ However, neither Cys239 nor Phe215 is positionally close to the catalytically active pocket of CYP3A4^5^ (Figure 1b). This raises a key question of how DQR unbinds from the catalytically active center and how the covalent bond is formed during this process. The answer to this question can help unravel the abnormal mechanism of suicide inhibition of CYP3A4 by raloxifene, which is however very difficult to obtain from experiments or available crystal structures.

To address this question, we resorted to computer simulation approaches to study in detail the unbinding process of DQR from the catalytically active center of CYP3A4 and thereby reveal the mechanism of suicide inhibition of CYP3A4 by raloxifene. Notably, as mammalian CYPs are membrane-attached enzymes, our simulations were carried out for CYP3A4 in the presence of a lipid bilayer.^10^ We incorporated the Ratchet&Pawl potential (RPP) into potential scaled molecular dynamics (sMD) simulations, which are hereafter called sMD–RPP simulations, to efficiently generate the trajectories used for identifying the potential paths for DQR unbinding from CYP3A4. For each of such potential paths, we built a “guess path” for the path collective variable (CV) and applied metadynamics simulations to construct the free-energy surface (FES) for the DQR unbinding process and identified the key intermediate states. We show that one of the key intermediate states in the DQR unbinding process is stabilized by a covalent bond formed between DQR and Cys239 of CYP3A4. In this intermediate state, DQR also stabilizes the hydrophobic cavity formed due to the expansion of the F′ and G′ helices and blocks the passage of the substrate.

## Results

### Determination of Guess Paths

**Figure 2:**
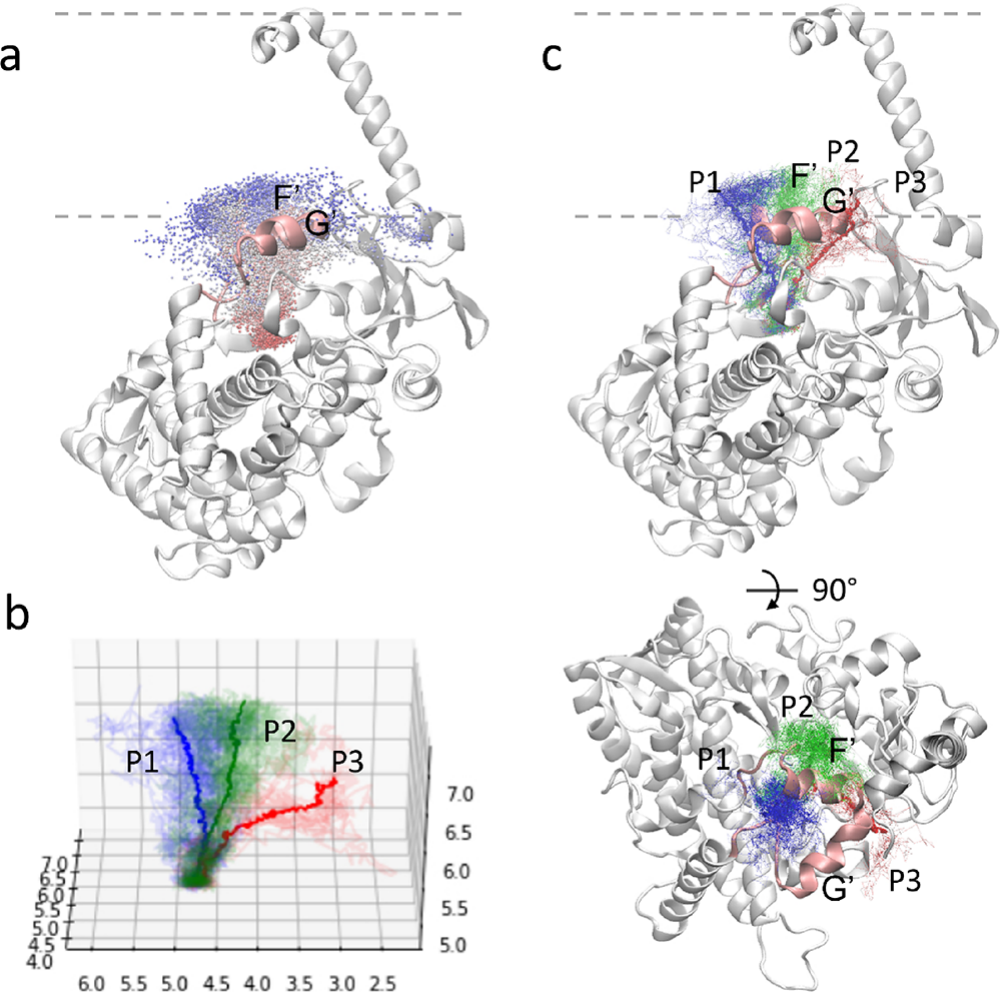
Unbinding trajectories obtained from the sMD–RPP simulations and the “guess paths.” (a) The unbinding trajectories from 50 sMD–RPP simulations, represented by the evolution of the DQR COM from *t* = 0 (in red) to the unbound state (in blue). The protein is shown in cartoon and the membrane surface in dashed line. (b) The streamlines of the COM of DQR and the centroids of the streamlines for each cluster. The *x*, *y*, and *z* axes represent the coordinates of the COM of DQR. (c) The three clusters for the unbinding paths and the ligand unbinding streamlines.

To disclose the DQR unbinding paths, we carried out in total 50 replicas of sMD–RPP simulations. Each simulation was stopped once DQR left the protein and entered the membrane. As shown in Figure 2, the location of DQR leaving the protein is close to the F′ and G′ helices where CYP3A4 is in contact with the membrane. The trajectories from the sMD–RPP simulations were then cleaned up, and the center of mass (COM) of DQR in each cleaned-up trajectory was represented as a streamline. Using cluster analysis, we identified three unbinding paths, P-4, P-2a, and P-2f, following the classification by Cojocaru et al.^11^ (Figure 2c). In the P-4 path, DQR left the protein from the middle of the F′ and G′ helices. In the P-2a path, DQR egressed from the protein via the region between the F′ and A helices. In the P-2f path, the egressed portal is in the region between the F′–G′ loop and the β1 turn.

During the process of DQR leaving the catalytic center of CYP3A4, the protein also underwent conformational changes, as observed from the sMD–RPP simulations. When DQR was leaving the catalytic center along the P-4 path, the angle between the F′ and G′ helices increased, forming a space so that DQR can pass through it. Thereafter, the angle between the two helices contracted (Figure S2). In the P-2a path, during the unbinding of DQR, the F′ and G′ helices moved upward and approached to the membrane, and the F′–F loop reversed. When DQR dissociated along the P-2f path, the F′ and G′ helices opened up and then approached to the membrane.

### Free Energy Profile for the Unbinding Paths

In order to obtain the energetics for DQR unbinding from the protein CYP3A4, we selected path CVs^12^ for the metadynamics simulations to construct the FES for the unbinding paths. We analyzed the positional change of DQR and protein conformational changes in the trajectories for each of the three unbinding paths, P-4, P-2a, and P-2 f. For each unbinding path, we built a guess path for the path CVs. *S*_path_ and *Z*_path_ were used to describe the position of a point in the configurational space with respect to the reference path, with *S*_path_ describing the progression along the unbinding path and *Z*_path_ describing the distance from the guess path (see the “Method” section for details). The metadynamics simulation was run for 2 μs for constructing the FES, and as shown in Figure S3, the free energy profiles converged after 1.5 μs.

**Figure 3:**
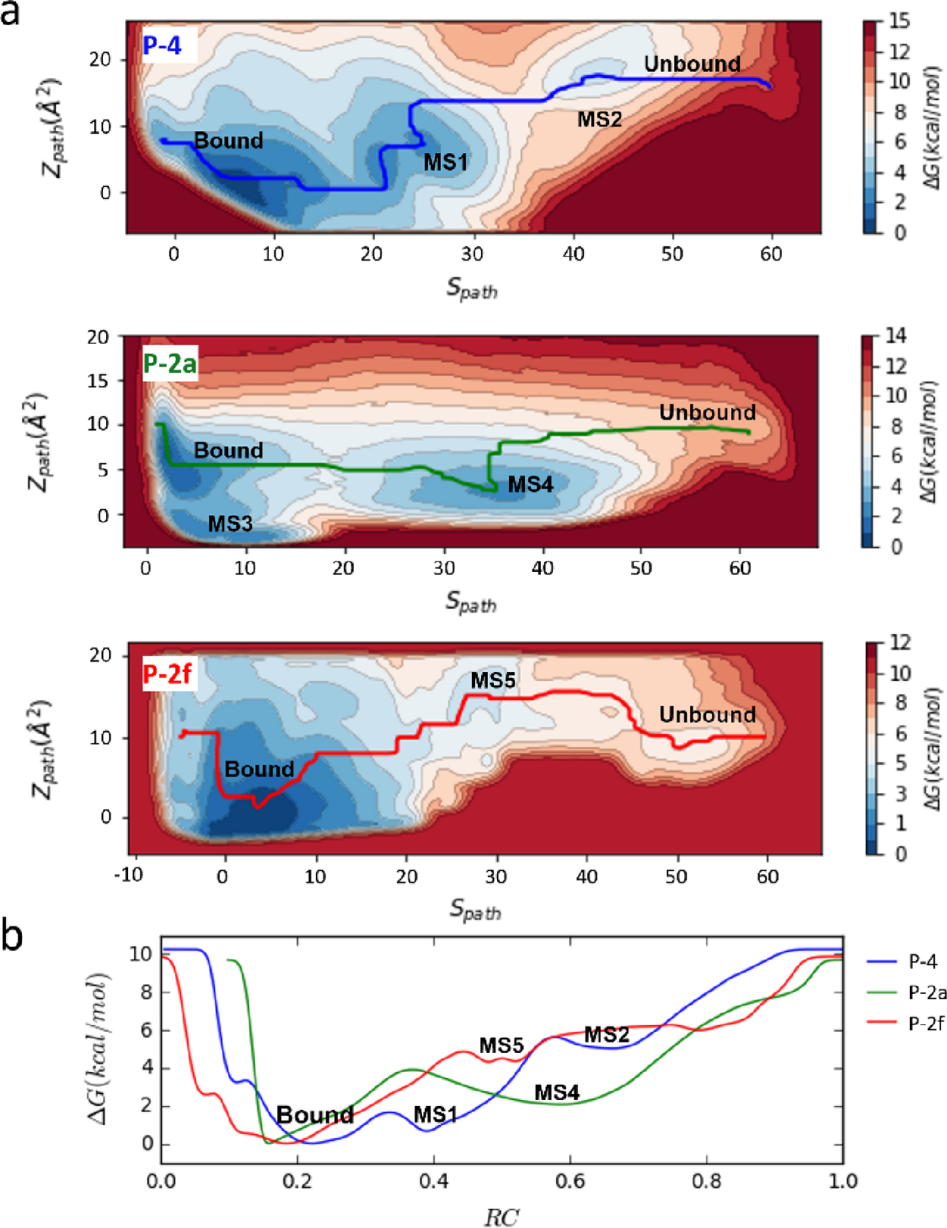
Free energy surfaces and the free energy profiles along the MFEPs. (a) Free energy surfaces for DQR unbinding from the CYP3A4 active center along the three unbinding paths (from top to bottom: P-4, P-2a, and P-2f). On each FES, the MFEP is displayed in solid line. (b) Free energy profile along each MFEP. For a better comparison, we normalized the reaction coordinate for each path and aligned the bound state at the lowest point of free energy.

From Figure 3, we can see that for each path, there is a deep minimum in the region with *S*_path_ around 0∼10, which corresponds to the bound state. Compared with the free energy near the region where DQR left the protein (*S*_path_ > 60), the free energy of the bound state is about −10 kcal/mol lower. Here, we would like to point out that the free energy changes are for DQR unbinding from the catalytic center along the unbinding paths and the unbound state corresponds to DQR in the membrane.

In addition to the deep minimum representing the bound state, several local minimums were also identified on each FES. On the FES for the P-4 path, there are two minimums, which are MS1 and MS2 near *S*_path_= 25 and *S*_path_= 40, respectively. Compared to the bound state, MS1 has a lower free energy of about 2.1 ± 0.3 kcal/mol, while MS2 has a higher free energy of about 6.5 ± 1.3 kcal/mol. There are also two minimums on the FES for the P-2a path, which are MS3 (*S*_path_ = 10) and MS4 (*S*_path_= 35). The *S*_path_ value for MS3 is very close to that for the bound state, but their *Z*_path_ values are different (the *Z*_path_ values are −12 and 0 Å^2^ for the bound state and MS3, respectively). The free energy for MS3 (1.0 ± 0.1 kcal/mol) is slightly higher than the bound state, and the free energy difference between MS4 and the unbound state is about 8.2 ± 0.1 kcal/mol. On the FES for the P-2f path, only one minimum MS5 (with *S*_path_ = 28 and *Z*_path_= 5 Å^2^) was found with a free energy of about 5.2 ± 0.7 kcal/mol. Among these intermediate states, MS1 on P-4 and MS4 on P-2a have lower free energies, while the free energies for MS2 on P-4 and MS5 on P-2f are relatively high. Thus, the intermediate states MS2 and MS5 may not be as stable as MS1 and MS4.

**Figure 4:**
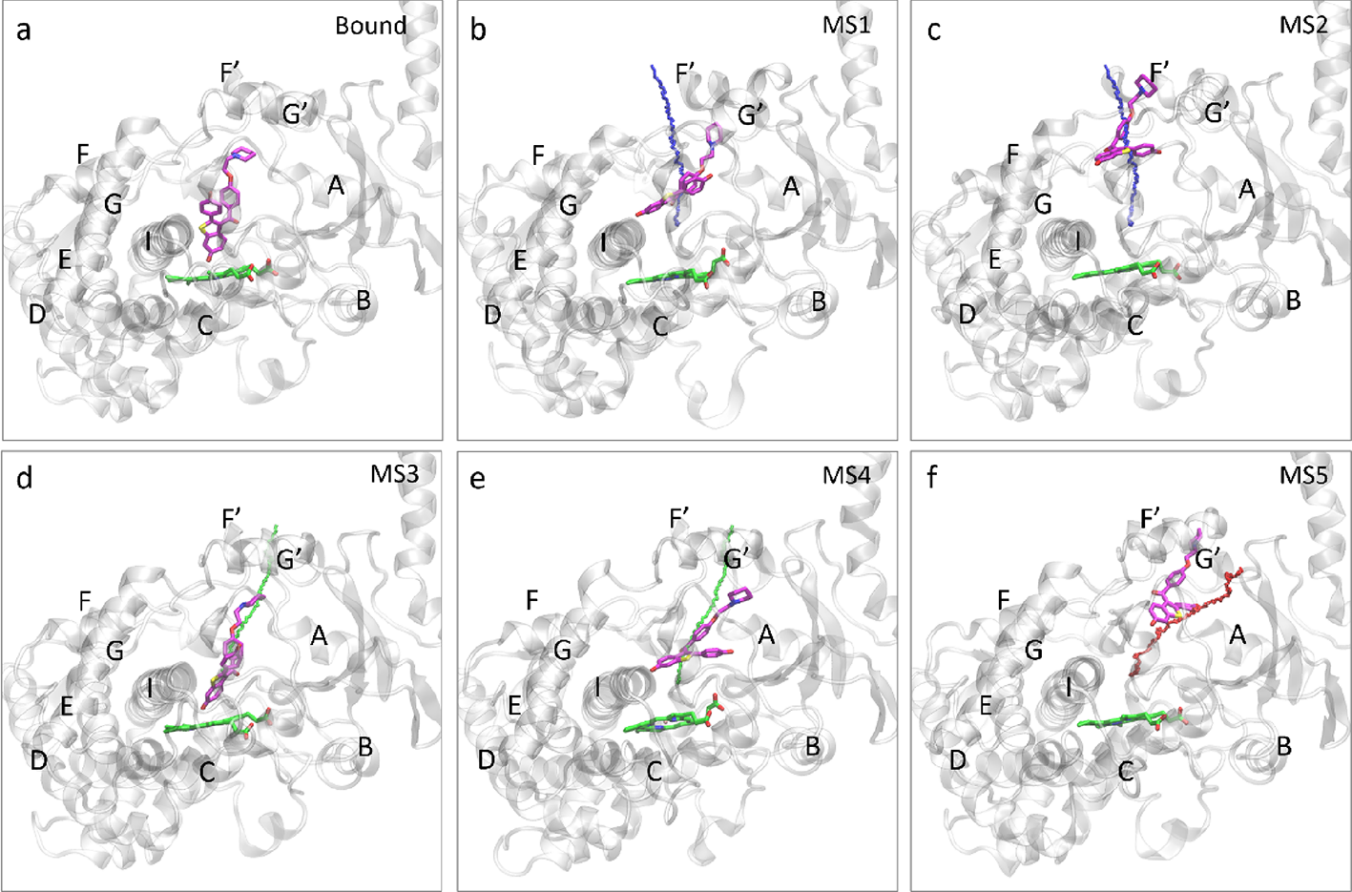
Representative structures for the bound state and metastable states on the FESs. (a) The representative structure for the bound state. (b–f) The representative structures for intermediate states MS1, MS2, MS3, MS4, and MS5, respectively. The protein is shown in cartoon. The ligand and heme are shown as sticks, with the ligand in magentas and heme in green. The positional change of the ligand COM in each guess path is indicated by dots, with blue, green, and red representing P-4, P-2a, and P-2f, respectively.

We further examined the representative structures for the intermediate states. As shown in Figure 4, DQR is close to the heme group in MS1 (*S*_path_=25), while in MS2 (*S*_path_=40), it is located near the egress portal of the protein. In MS3 (*S*_path_=10), the interaction between the oxygen on the benzothiophene ring of DQR and the heme group is kept as in the bound state and DQR has only rotated a little as compared to the bound state (Figure 4a). The location of DQR in MS4 (*S*_path_= 35) is close to that in MS1, with the root mean square deviation (RMSD) for DQR close to 3 Å after aligning the Cα atoms of the protein in the two states. In both states, DQR is away from heme and its aromatic rings are parallel to heme. The difference between the two states was found in the protein conformations (Figure S4). In MS1, DQR passes P-4 through a space opened by the F′ and G′ helices. In MS4, DQR passes P-2a through the space in between the F′ and A helices.

### Minimum Free Energy Paths Associated with the Unbinding Process

Here, we first study the difference between the guess path and the minimum free energy path (MFEP) on each FES. The MFEP was calculated with the MEPSA algorithm^13,14^ using the bound state (*S*_path_= 0, *Z*_path_= 0) as the starting point and the unbound state (*S*_path_= 60, *Z*_path_= 0) as the end point. As we can see from Figure 3, the guess path (where *Z*_path_ = 0) on each FES has some deviation from the MFEP, but overall the deviation is not large, indicating that the guess path essentially matches the MFEP. Since the guess path was obtained from the analysis of the trajectories produced by the sMD–RPP simulations, the result also indicates that although RPP was added, the MFEP can still be sampled with relatively high probability, which can be considered as a feature of sMD simulations.^15,16^

We have also studied the free energy profile along the MFEP on each FES (see Figure 3b). The free energy profile along the P-4 or P-2a path is relatively flat due to the presence of multiple local minimums. The MFEP for P-4 has two local minimums, MS1 and MS2. The energy barrier from the bound state to MS1 is about 2 kcal/mol, and that from MS1 to MS2 is about 6 kcal/mol. On the MFEP for P-2a, the energy barrier from the bound state to MS4 is 4 kcal/mol. Although the location of DQR in MS1 is close to that in MS4, the energy barriers from the bound state to the two states are different because the conformational changes of the F′ and G′ helices are different in the two paths. After passing through MS4, the system needs to cross an energy barrier of 6 kcal/mol to leave the binding pocket. Along the P-2f path, the free energy barrier is steeper and there is almost no local minimum except for the shallow MS5. During the dissociation of DQR through P-2f, the system has to continuously cross the energy barrier with a height of 10 kcal/mol.

From the analysis of the free energy profile of the MFEP on each FES, we can conclude that P-4 and P-2a are energetically more favorable than P-2f for DQR unbinding from CYP3A4. We have also analyzed the 50 unbinding trajectories generated from the sMD–RPP simulations and found that there are 19 unbinding trajectories along P-4, 23 along P-2a, and only 8 along P-2f. This indicates that DQR prefers to unbind from the protein along the P-4 or P-2a path. Thus, the results from the MFEP study are in good agreement with those observed from the sMD–RPP simulations.

Finally, we picked out some representative structures for the local minimums along the MFEPs and performed an unbiased MD simulation for each structure. The simulations show that the structures for MS1, MS2, and MS4 remain stable for at least 200 ns, respectively, as indicated by the low fluctuations of the RMSDs in Figure S5. The structure for MS5 was not stable in the unbiased MD simulation. This observation matches the fact that MS5 corresponds to a shallow energy minimum. We also calculated the binding free energy for each of these states using the free energy perturbation (FEP) method. Using the thermodynamic cycle shown in Figure S6, we calculated the free energy differences with respect to the unbound state corresponding to DQR in the membrane for MS1, MS2, and MS4, which were – 5.1 ± 1.4, −5.7 ± 1.6, and – 2.3 ± 1.5 kcal/mol, respectively. These states are not as stable as the bound state, which has the binding free energy of −9.8 ± 1.5 kcal/mol lower than the unbound state.

### Suicide Inhibition Revealed by the Intermediate States

**Figure 5:**
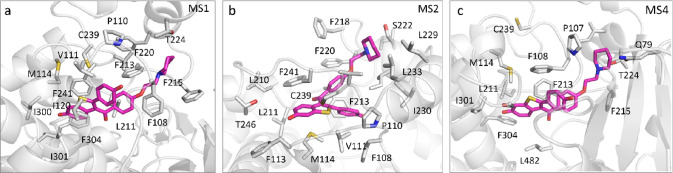
Representative binding modes for the relevant metastable states along the MFEPs.

Here, we examine the binding modes of DQR in MS1, MS2, and MS4. As shown in Figure 5, in the MS1 state, DQR is located below the F′ and G′ helices and wrapped in the hydrophobic environment formed by Phe108, Val11, Leu211, Phe215, Phe213, Phe220, Phe241, Ile300, Ile301, and Phe304. The benzothiophene ring of DQR is stabilized by forming pi–pi interactions with Phe304 and Phe241. In the MS2 state, DQR is wrapped in the hydrophobic environment formed by the F′ and G′ helices, which is composed of Pro110, Val111, Phe113, Met114, Leu210, Phe213, Leu233, Phe220, Leu229, and Phe241. In this hydrophobic environment, the benzothiophene and phenol rings of DQR form pi–pi interactions with Phe241 and Phe213, respectively, and the benzothiophene ring is close to Cys239. In the MS4 state, although the spatial position of DQR in the protein pocket is similar to that in the MS1 state, the F′ and G′ helices are not open as wide as in MS1 and the interaction patterns of DQR with the protein residues are therefore rather different. In the MS4 state, DQR is not in contact with the surrounding hydrophobic residues as tightly as in MS2. In the MS2 state, Phe108 on the BC loop is close to the benzothiophene ring and stabilizes DQR, while in the M4 state, Phe108 is located above the benzothiophene ring to hinder DQR from approaching Cys239. Due to the conformational difference of the BC loop, Cys239 is close to DQR in MS1, but not in MS4.

From the binding mode analysis, we note that DQR in the MS1 or MS2 state is in close contact with Cys239 of CYP3A4. In the MS1 state, the distance between the ortho-carbon of the DQR phenol ring and the sulfur atom on the thiol group of Cys239 is about 3.3 Å. In the MS2 state, the distance between the 7-position of the DQR benzothiophene ring and the sulfur atom of Cys239 is about 3.5 Å. In both states, DQR is rather close to Cys239 and is stabilized by the hydrophobic environment. Since Cys239 of CYP3A4 is highly related to the suicide inhibition of the enzyme during the biotransformation of raloxifene,^5,6^ we believe that MS1 and MS2 are the states in which DQR most likely forms a covalent bond with Cys239.

**Figure 6:**
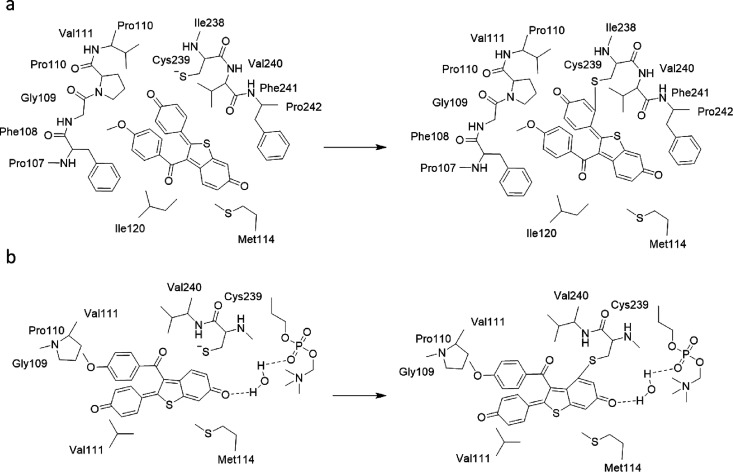
Schematic illustration of the reactions in MS1 (a) and MS2 (b).

In order to verify whether DQR can form a covalent bond with Cys239 in the two intermediate states, we performed a series of quantum chemistry (QC) calculations for the clusters representing the two states (see Methods). The results show that in MS1, the ortho position of the DQR phenol ring forms a covalent bond with Cys239, and in MS2, the 7-position of the DQR benzothiophene ring forms a covalent bond with Cys239 (Figure 6). In MS2, when DQR forms a covalent bond with Cys239, the benzothiophene ring further extends into the area between the F′ and G′ helices, and the oxygen on the 6-position of the benzothiophene ring forms a water-mediated hydrogen bond with the phosphate oxygen on a lipid molecule, which further stabilizes DQR and the surrounding residues.

## Discussion

Previous studies show that unbinding of a ligand from its protein receptor is not limited through one path.^17−19^ From our study of the unbinding mechanism of DQR from CYP3A4, we found that DQR can egress from the protein mainly via two paths, through the region either between the F′ and G′ helices (P-4) or between the F′ and A helices (P-2a). The path between the F′–G′ loop and the β1 turn (P-2f) has a higher energy barrier and is not among the most likely paths. It is also noticed that the preliminary sMD–RPP simulations indicated that the unbinding of DQR along P-4 has almost the same probability as along P-2a. This reflects that sMD–RPP can sample the MFEP with relatively high probability and provide useful information about an unbinding process.

The conformational changes of the protein play an important role in the unbinding process of DQR from the protein. Along the P-4 path, when the F′ and G′ helices are open, an extra space between the two helices is formed to allow DQR to pass through the space. On this path, two key intermediate states, MS1 and MS2, were identified, in which DQR is stabilized by the hydrophobic and pi–pi stacking interactions with the surrounding residues. When DQR unbinds along the P-2a path, the F′ and G′ helices are not open as wide as in P-4 but lift toward the membrane to allow DQR to pass through the space between the F′ and A helices. One intermediate state, MS4, was identified on this path.

Although the spatial positions of DQR in MS1 and MS4 look similar, the binding modes of DQR in the two states are different. In the MS4 state, the F′ and G′ helices are not open as wide as in MS1 or MS2 and the free energy of this state is higher than those of MS1 and MS2. Phe108 prevents DQR from entering the hydrophobic cavity and approaching Cys239. In the MS1 and MS2 states, DQR can enter the cavity because of the opening of the F′ and G′ helices and thus has the chance to approach Cys239. For this reason, only P-4 can be used to explain the mechanism of the covalent binding interaction between DQR and Cys239 of CYP3A4. The QC calculations show that in the MS2 state, the 7-position of the DQR benzothiophene ring forms a covalent bond with the sulfur atom of Cys239. Since DQR is located close to the egress portal, the formation of the covalent bond blocks small molecules from entering or leaving the protein, leading to the inactivation of the enzyme. Thus, our study explains in detail the suicide inhibition phenomenon observed in the experiment, which can hardly be revealed by the mutagenesis or mass spectrometry experiments. Our results also indicate a testable hypothesis: if DQR inhibits the enzyme by blocking a particular path (e.g., P-4), the inhibition of substrates that use different paths (e.g., channels 1, 2e, and S) might be less pronounced. Experiments could be performed for metyrapone (egress through channel 1) and ritonavir analogues (egress through channel S) with/without DQR.^20−22^ As DQR is bulky, it may block the activity of the enzyme regardless of the existence of alternative pathways, and we would also like to suggest to use smaller substrates to test this hypothesis.

We revealed that the DQR unbinding process and suicide inhibition of CYP3A4 by raloxifene are closely related to the conformational changes of CYP3A4. Protein conformational changes have also been observed during the caffeine metabolite unbinding from CYP3A4.^23^ In such a case, if the bias is applied only on the ligand, such as the metabolite, it is difficult to model correctly the unbinding process. In an sMD–RPP simulation, the potential of the system is scaled down uniformly. This reduces the energy barriers for protein conformational changes and the ligand unbinding process. As such, the ligand unbinding process in the sMD–RPP simulation is greatly accelerated. Although the path observed from an sMD–RPP simulation may deviate from those for a real unbinding process, it can serve as a guess path for metadynamics to help build the FES for the unbinding process and find out the MFEP. We have successfully applied this methodology to the study of the unbinding process of DQR from CYP3A4 and clarified the suicide inhibition mechanism.

## conclusions

In this work, through combining enhanced sampling simulation and QC calculation approaches, we successfully revealed the unbinding mechanism of the raloxifene metabolite DQR from the catalytic center of CYP3A4. The mechanism explains well the abnormal suicide inhibition phenomenon of CYP3A4 during the raloxifene biotransformation process, which is difficult to understand from the crystal structure of the bound complex or conventional MD simulations. Our work highlights the importance of protein conformational changes in the metabolite unbinding process. The methodology presented here can also be used to reveal in atomic detail the ligand unbinding mechanism for a system where the ligand is deeply buried in the protein and the unbinding process is strongly coupled to protein conformational changes.

## Methods

### System Preparation

The major reactive product of raloxifene, raloxifene DQR, was prepared by LigPrep (Schrödinger, LLC, New York, 2018). Molecular docking was carried out using Glide for predicting the initial binding mode of DQR to the active site of CYP3A4. As the catalytic site of CYP3A4 shows large plasticity,^24,25^ we carried out ensemble docking to select the catalytically feasible pose for DQR. Eleven CYP3A4 crystal structures, with the PDB codes 1TQN, 2V0M, 3NXU, 3UA1, 4D78, 4I4G, 4K9T, 4K9V, 4K9W, 5TE8, and 5VC0, respectively, were selected for docking. In the preparation of these structures, the co-crystalized ligand and water molecules were removed. The missing residues were added, and the protonation states of the ionizable residues were determined by the Protein Preparation Wizard (Schrödinger, LLC, New York, 2018). The Cpd I form of the heme moiety was not considered in docking and MD simulations. The center for docking was located at the point 5 Å from the heme iron, and the residues within 20 Å of the docking center were involved in docking. Flexible side chain docking was not considered. For each docking run, ChemScore with the scoring template parameterized for heme-containing proteins was used to rank the 50 output poses generated by the genetic algorithm. The crystal structure that can generate the near-attack conformation of DQR was selected for further preparation for MD simulations.

The system for MD simulations was prepared as follows. Since the human CYP3A4 enzyme is a membrane-anchored protein, we first constructed a full-length model of CYP3A4. The missing N-terminal residues were modeled using the MODELLER program.^26^ Thereafter, the CYP3A4–DQR complex thus prepared was embedded into a 1-palmitoyl-2-oleoyl-sn-glycero-3-phosphocholine (POPC) bilayer membrane of 256 POPC molecules, which was generated from the CHARMM-GUI web server.^27^ VMD was used for embedding the protein to the POPC membrane, with the membrane position predicted by the OPM web server.^28^ Lipid molecules within 0.9 Å of the protein were deleted, which reduced the number of POPC molecules to 242. The orientation of the enzyme on the membrane was modeled by referring to previous works.^29,30^ The heme tilt angle with respect to the membrane normal was 73.2° (Figure S1). The system was further solvated and neutralized to meet the condition with the concentration of NaCl being 0.15 M. This was implemented by adding 21,062 water molecules, 110 sodium ions, and 114 chloride ions into the system. The AMBERff99SB-ILDN force field, general amber force field (GAFF), and Slipids force field were used for the protein, DQR, and membrane molecules, respectively.^31−33^ The force field parameters for heme were obtained from Shahrokh et al.’s work.^34^ Restrained electrostatic potential (RESP)-derived charges were assigned to DQR based on the electrostatic potential derived from the Hartree–Fock calculation at the HF/6-31G(d) level, in which the DQR geometry optimized at the density functional theory (DFT) B3LYP/6-31G(d) level was used. Before MD simulations, energy optimization was carried out for the system. The steepest descent method was first carried out with harmonic restraints on the non-water atoms, the protein heavy atoms, and the main chain atoms of the protein, respectively. The final minimization step was accomplished using the conjugate gradient method without any restraint. A 200 ps restrained MD simulation in the NVT ensemble (with *T* = 300 K) was carried out for the system, followed by a 500 ps simulation carried out in the NPT ensemble (with *T* = 300 K and *P* = 1 atm). Thereafter, a long-time simulation of 1000 ns was performed for equilibration, during which no restraint was applied. All the simulations were performed with GROMACS 2018 patched with Plumed 2.3.^35−37^ The cutoffs for the short-range electrostatic interactions and van der Waals interaction were set to 10 Å. The particle-mesh Ewald (PME) method was used to recover the long-range electrostatic interaction.^38^ The LINCS algorithm was used to constrain the bonds involving hydrogen atoms.^39^ A time step of 2 fs was used in all the simulations.

### Generation of Preliminary Unbinding Trajectories

To generate the preliminary DQR unbinding trajectories, we exploited sMD in combination with the RPP.^40,41^ sMD and RPP have been successfully used to study ligand unbinding processes, respectively.^15,42^ In an sMD simulation, the potential of the system is scaled uniformly by a scaling factor β. For an unbinding process that is strongly coupled to the protein conformational changes, sMD can help disclose the hidden degrees of freedom closely related to the conformational changes. However, because DQR is deeply buried into the protein, it is difficult to use sMD alone to simulate the ligand unbinding event. Inspired by the work of Capelli et al., which used RPP to generate the unbinding trajectories,^42^ we introduced RPP into our sMD simulations. The RPP is defined as
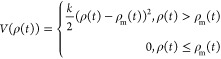
with
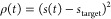
and
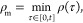
where *s*(*t*) is the ratcheting coordinate defined as the projection of the COM distance between DQR and heme on the direction normal to the membrane. The bias becomes zero when DQR is away from the binding site and damps the fluctuation when it moves in the opposite direction. We call this methodology, in which the RPP potential is introduced into sMD, sMD–RPP. We found that in our sMD–RPP simulations, only a small force constant *k* is needed to induce the dissociation of the ligand. From a series of test simulations, we found that the combination of *k* = 0.024 kcal/mol/Å^4^ and β = 0.6 is appropriate for using sMD–RPP simulations to disclose the protein conformational changes in the ligand unbinding process.

### Identification and Construction of Guess Paths

The guess paths for the ligand unbinding process were identified from the 50 unbinding trajectories generated from the sMD–RPP simulations. First, the trajectories from the bound to the unbound state were processed in order to filter out all the non-productive configurations such as detours, dead-ends, and loops that occurred in systems whose dynamics was not in detailed balance.^15,43^ The trajectories from the bound to the unbound state were cleaned up as described by Schuetz et al.^15^ All the frames were scanned by comparing the RMSDs after the alignment of the α carbon atoms of the protein. A scanning frame was saved when its RMSD with respect to the last saved frame was greater than or equal to a given threshold (3 Å). Thereafter, the trajectories of the COM of the ligand were treated as streamlines. The streamlines were clustered by QuickBundles,^44^ which is an algorithm for clustering and merging similar streamlines with common centroids. From visual inspection, QuickBundles could cluster the unbinding trajectories into three different types. For each cluster, the frames from the cleaned-up trajectories were scanned and those with COM close to the centroid were selected and clustered to represent the structures that frequently occurred. For these structures, additional Ratchet&Pawl MD simulations were carried out to generate the configurations between each pair of endpoints as inspired by Bernetti et al.^45^ This procedure thus generated a large number of frames along a guess path, allowing us to select the frames that were equally spaced, which is a crucial requirement for constructing the path CVs to be discussed in the next section.

### Metadynamics and Path CVs

Metadynamics^46,47^ was used in this study. In a metadynamics simulation, a history-dependent bias potential is added over a set of selected CVs, with
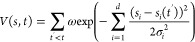
where ω is the height and σ_*i*_ is the width of the bias potential deposited over the *i*th CV. Following the method introduced by Branduardi et al.,^12^ path CVs are used to describe the essential conformational changes along an unbinding process. The progression along the reference path (*S*_path_) and the deviation from the reference path (*Z*_path_) are defined as
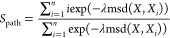
and
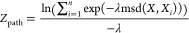
where *X* denotes the coordinates of interest at the current simulation time step, *X_i_* denotes the coordinates of the *i*th reference frame that composes the path, msd(*X*, *X_i_*) is the mean square deviation between *X* and *X_i_*, and λ is a smoothing parameter. With this definition, *S*_path_ takes the value from 1 to N (i.e., the total number of reference frames) and represents the progress along the unbinding coordinates, and *Z*_path_ increases monotonically as the system moves away from the reference path.^48^

In our study, msd(*X*, *X_i_*) between the heavy atoms of DQR and the Cα atoms of the F–G loop (Lys209 to Arg243) was calculated after the alignment of the rest of the Cα atoms of the protein (Val71 to Arg207, Glu244 to Glu362, and Gly444 to Gln463). The reference coordinates were selected from the cleaned-up unbinding trajectories with the criteria that the frames were equally spaced.^12^ In this work, we used the well-tempered metadynamics simulation method.^49^ GROMACS patched with Plumed 2.3 was used for the metadynamics simulations. The initial Gaussian height was 0.2 kcal/mol, and the bias factor was 15. The Gaussian widths were 0.1 and 0.01 nm^2^ for the biases on *S*_path_ and *Z*_path_, respectively. The bias potential was added every 2 ps.

### FEP

FEP was used to estimate the binding free energy of DQR in the intermediate states. The calculations were carried out in line with our previously work.^50^ As designed according to the thermodynamics cycle (Figure S6), the perturbation path was built to decouple the van der Waals and electrostatic interactions between DQR and CYP3A4, with 20 λ windows (0.05, 0.1, 0.15, 0.2, 0.25, 0.3, 0.35, 0.4, 0.45, 0.5, 0.55 0.6, 0.65, 0.7, 0.75, 0.8, 0.85, 0.9, 0.95, 1.0) for decoupling the van der Waals interaction and 11 λ windows (0.0, 0.1, 0.2, 0.3, 0.4, 0.5, 0.6, 0.7, 0.8, 0.9, 1.0) for annihilating the electrostatic interactions, respectively. During the decoupling, soft-core potentials were used.^51^ The soft-core parameter (sc_alpha) and soft-core power (sc_power) were set to 0.5 and 1.0, respectively. The radius of interaction (sc_sigma) was set to 3.0 Å. For each window, energy minimization and two sequential 200 ps equilibration simulations (using the NVT ensemble at 300 K and NPT ensemble at 300 K and 1 atm, respectively) were carried out before the production run of 10 ns. The relative position of DQR with respect to CYP3A4 was restrained via the harmonic potentials on one distance, two angles, and three dihedrals with the force constant of 10 kcal mol^–1^ Å^–2^/10 kcal mol^–1^ deg^–2^. The contribution of the restrains to the free energy including the standard state correction was calculated analytically as described by Boresch et al.^52^ The free energies were calculated with the Bennet acceptance ratio method provided in pymbar.^53^

### QC Calculations

Based on the binding modes in the MS1 and MS2 states, we constructed two cluster models, namely, cluster MS1 and cluster MS2, for QC calculations. DQR was truncated at the C–C bond between the 2-piperidin and phenol groups in both models. The protein residues Pro107–Val11, Met114, Ile120, and Cys239–Phe241 were included in the MS1 cluster, and the residues Pro110, Val111, Met114, Cys239, and Val240 and one lipid molecule were included in the MS2 cluster. The net charge for each model was −1, while the numbers of atoms were 213 and 202 for the MS1 cluster and MS2 cluster, respectively. To avoid unrealistic movement, some atoms were fixed during the energy optimizations, as depicted in Figure S7.

The Gaussian 09 (Rev. E01) program was used for the QC calculations of the reactants and the covalent adducts in the intermediate state revealed by metadynamics. There is no base residue surrounding Cys239, and we therefore did not carry out a transition state search of the hydrogen abstraction and assumed that the formation of the sulfhydryl anion is caused by the surroundings. The B3LYP hybrid exchange-correlation functional, together with Grimme’s empirical method for recovering the dispersion energy, was used for all the calculations, including geometry optimizations. The geometry optimizations and single-point (SP) energy calculations were carried out at the same level of theory with the 6-31G(d,p) and 6–311++G(2d,2p) basis sets, respectively. SP energy calculations were also carried out with the SMD solvation model using the dielectric constant ε = 4 and corrected with the zero-point energies (ZPEs), which were obtained from the frequency calculations with the same basis set as for the geometry optimizations.

## Notes

The PDB files used in this study were downloaded from the RCSB protein data bank (https://www.rcsb.org/). Information for the software used in this study is as follows: Schrödinger, LLC, New York, NY, 2018; MODELLER 9.9; CHARMM-GUI web server (https://www.charmm-gui.org/); VMD (1.9.2); GROMACS 2018 patched with Plumed 2.3; pymbar (http://github.com/choderalab/pymbar); Gaussian 09 (Rev. E01). The parameter settings in the simulations and the detailed descriptions can be found in the “Methods” section and Supporting Information.

## Supporting Information Available

Model of CYP3A4 anchored in membrane and the tilt angle of heme; representative conformational changes of the F′ and G′ helices; convergence analysis for the metadynamics simulations; protein conformations in the MS1 and MS4 states; evolution of RMSD for DQR in the metastable state; representation of the thermodynamic cycle; cluster models for MS1 and MS2; reaction energies from QC calculations; and cartesian coordinates of the clusters (PDF)
