# Correction to “Unraveling the Abnormal Molecular Mechanism of Suicide Inhibition of Cytochrome P450 3A4”

**DOI:** 10.1021/acs.jcim.3c00233

**Published:** 2023-03-07

**Authors:** Yang Zhou, Junhao Li, Glib Baryshnikov, Yaoquan Tu

In our original article (https://pubs.acs.org/doi/full/10.1021/acs.jcim.2c01035),Figure 6 and Table S1 show the cluster modes used in the quantum chemistry calculations. Unfortunately, Figure 6 and Table S1 contain errors regarding the ligand bonding site, double bonds in the ligand, and labels of the surrounding residues, and the positions of the constraints were not given in Figure 6 nor in Table S1. To avoid misleading the reader about our simulations, here, we show the correct Figure 6 and Table S1.

**Figure 6:**
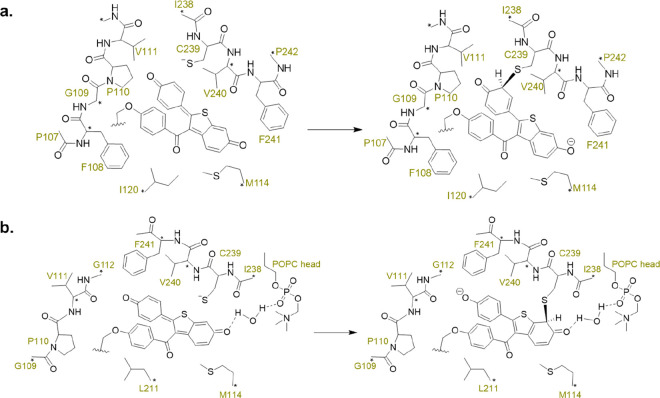
Schematic illustration of the QC cluster models MS1 (a) and MS2 (b). The protein residues (and membrane component in MS2) are labeled in a light color, while the frozen atoms are marked with pentacles.

**Table S1:**
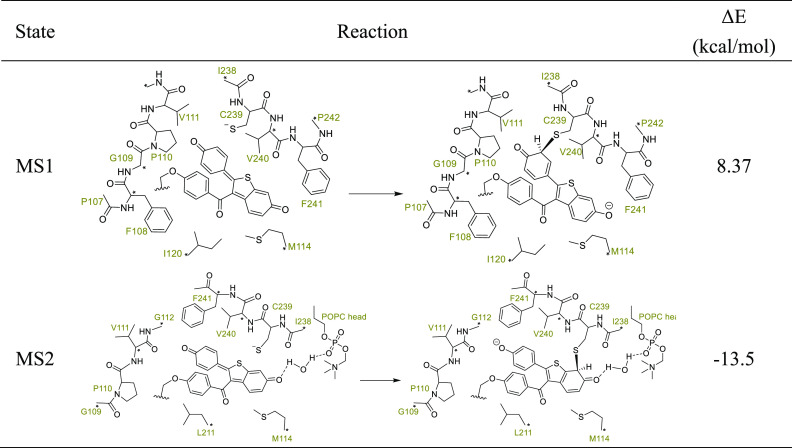
Reaction energies for the formation of the covalent bond between DQR and Cys239 of CYP3A4 in the MS1 and MS2 states obtained from quantum chemistry calculations. Δ*E* values were calculated at the B3LYP-D3(BJ)/6-311++(2d,2p)/PCM(SMD)/ZPE level.

