# Supplementary material for: Unraveling the Abnormal Molecular Mechanism of Suicide Inhibition of Cytochrome P450 3A4
Source: J Chem Inf Model. 2022 Dec 2;62(23):6172–81. doi: 10.1021/acs.jcim.2c01035 (PMC9749025; doi:10.1021/acs.jcim.2c01035)
Supplement: Supplementary file 1 — ci2c01035_si_001.pdf [file ci2c01035_si_001.pdf]

Supporting Information

Unraveling the Abnormal Molecular Mechanism of Suicide Inhibition of Cytochrome P450 3A4

Yang Zhou[a,b], Junhao Li[b], Glib Baryshnikov[c], and Yaoquan Tu[b*]

[a]School of Pharmacy, Jinan University, 601 Huangpu Avenue West, Guangzhou 510632, China
[b]Department of Theoretical Chemistry and Biology, KTH Royal Institute of Technology, 114 28 Stockholm, Sweden
[c]Laboratory of Organic Electronics, Department of Science and Technology, Linköping University, 60174 Norrköping, Sweden

* Corresponding author:
Yaoquan Tu, Department of Theoretical Chemistry and Biology, KTH Royal Institute of Technology, 114 28 Stockholm, Sweden
Tel.: +46 8 790 96 45
Email: Y. Tu (yaoquan@kth.se)

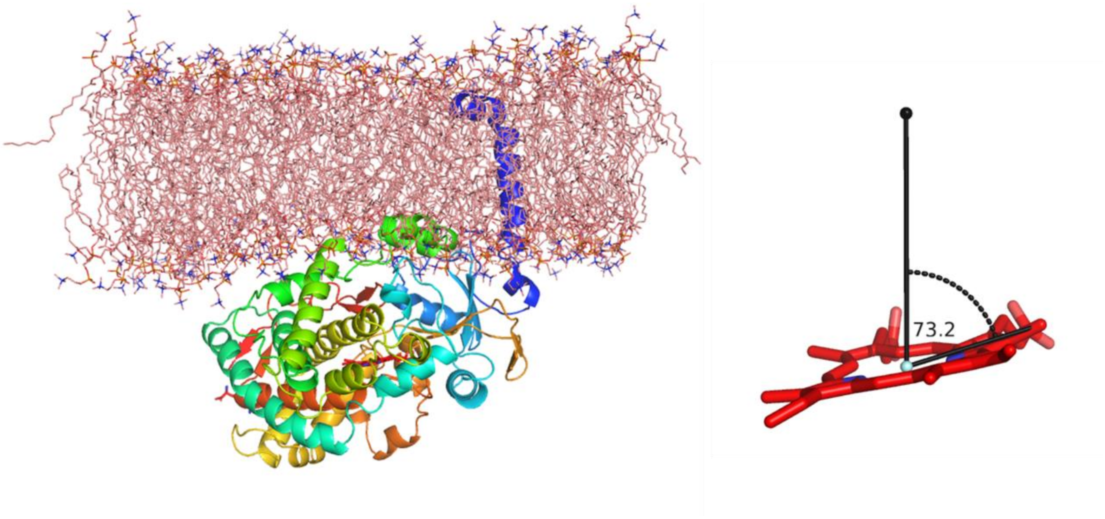

Figure S1. Model of CYP3A4 anchored in membrane and the tilt angle of heme.

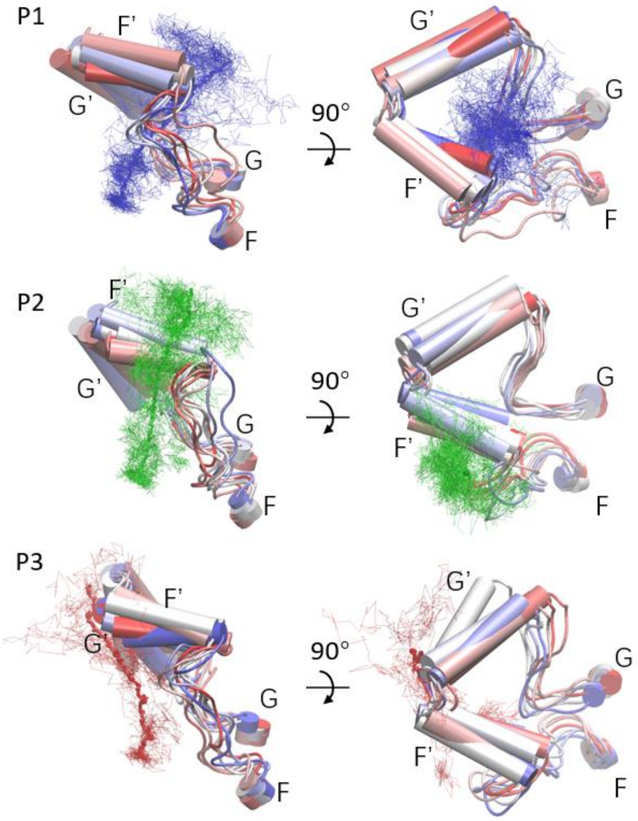

Figure S2. Representative conformational changes of the F' and G' helices of the protein CYP3A4 along the three potential paths, P1, P2, and P3. The helices are shown in cartoon with the conformations in the bound and unbound states shown in red and blue, respectively.

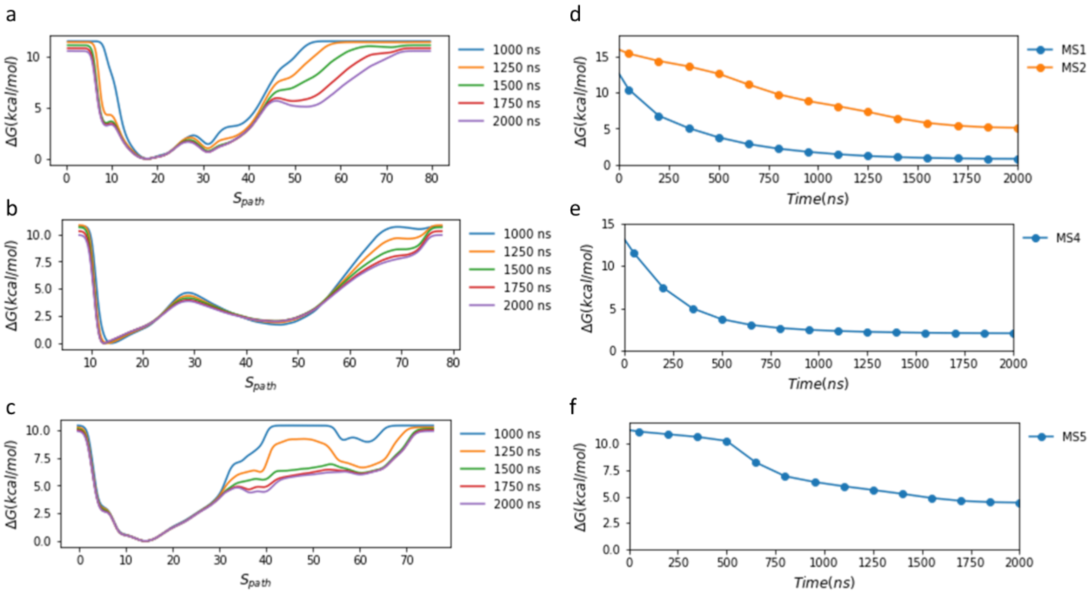

Figure S3. Convergence analysis for the metadynamics simulations. (a-c) Free energy profiles of P-4 (a), P-2a (b), and P-2f (c), obtained from different simulation times. (d-e) Changes of the free energy differences between the metastable states and the bound state with the simulation time.

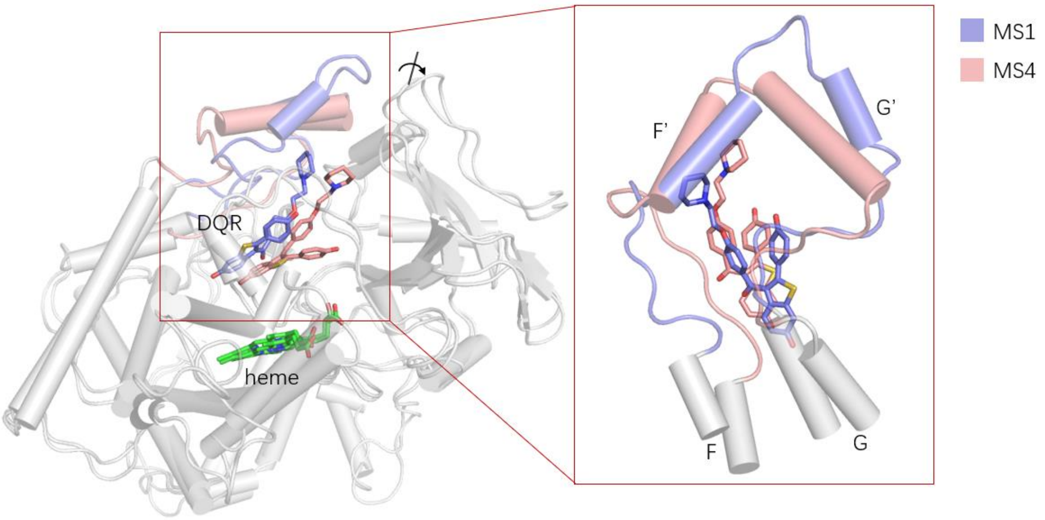

Figure S4. Protein conformations in the MS1 and MS4 states.

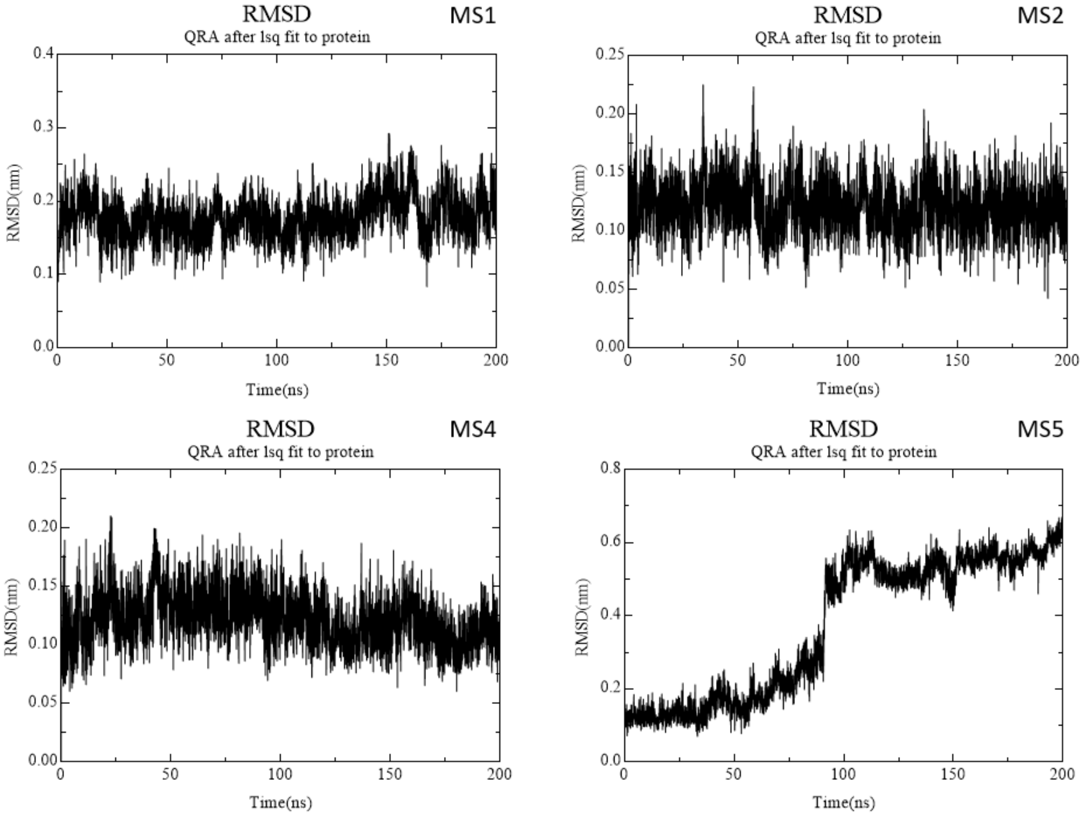

Figure S5. Evolution of RMSD for DQR in states MS1, MS2, MS4, and MS5 after the protein was least-square fitted to its initial structure.

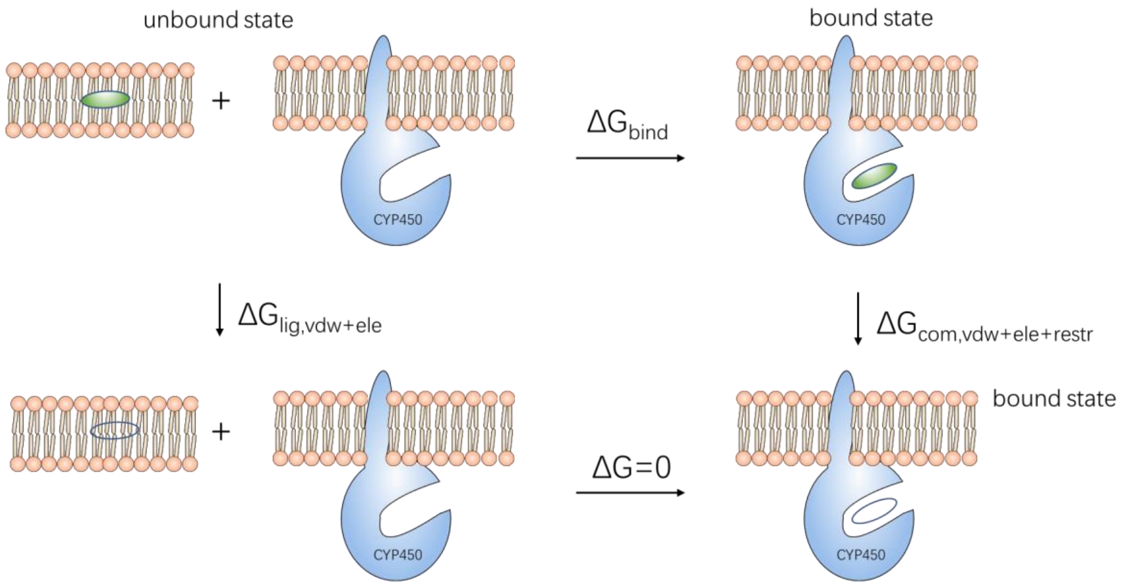

$$\Delta G_{bind} = -\Delta G_{com,vdw+ele+restr} + \Delta G_{lig,vdw+ele} - \Delta G_{restr}$$

Figure S6. Schematic representation of the thermodynamic cycle for free energy calculations.

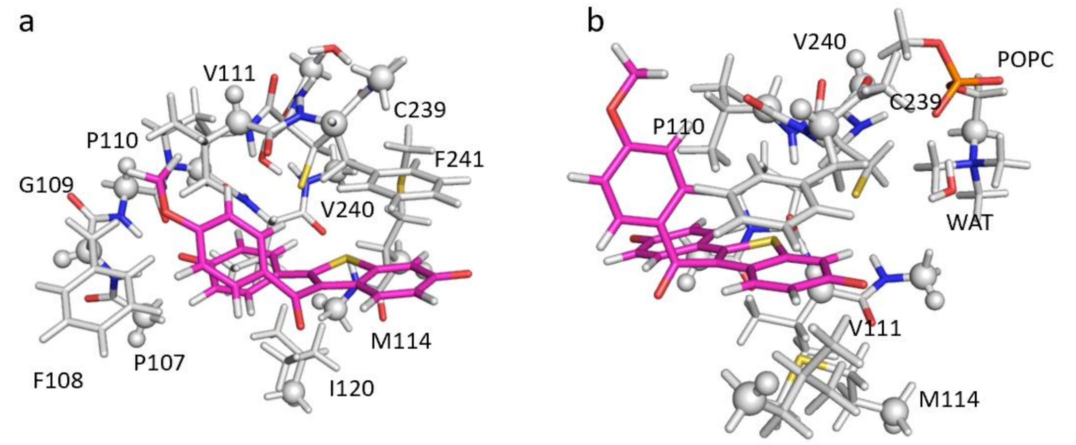

Figure S7. Cluster models for MS1 (a) and MS2 (b). The atoms fixed during the energy optimizations were shown as spheres.

Table S1. Reaction energies for the formation of the covalent bond between DQR and Cys239 of CYP3A4 in the MS1 and MS2 states obtained from quantum chemistry calculations. ΔE values were obtained from the quantum chemistry calculations carried out at the B3LYP-D3(BJ)/6-311++(2d,2p)/PCM(SMD)/ZPE level.

State	Reaction	ΔE (kcal/mol)
MS1	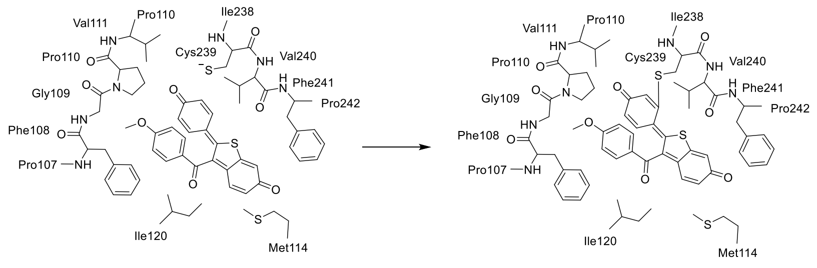	8.37
MS2	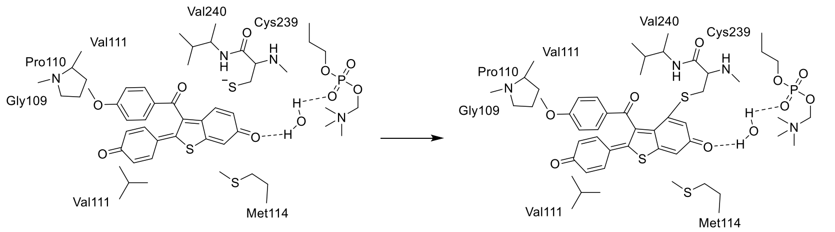	-13.5

Cartesian coordinates of the clusters

MS1

C	5.856614	0.016676	3.097997
H	5.199592	-0.844122	2.967526
C	6.880261	0.176844	1.968764
O	7.934786	0.784186	2.124425
N	6.523105	-0.409392	0.789309
H	5.554305	-0.720041	0.716289
C	7.199561	-0.154164	-0.461005
H	8.282736	-0.084157	-0.360784
C	6.807093	-1.235444	-1.501276
H	7.450344	-1.096104	-2.373985
H	5.780476	-1.031982	-1.828144
C	6.875392	-2.643714	-0.968478
C	5.698876	-3.357446	-0.713804
H	4.739292	-2.897460	-0.930959
C	5.742171	-4.640499	-0.167839
H	4.814923	-5.169431	0.026512
C	6.970513	-5.233880	0.121726
H	7.008831	-6.232165	0.547844
C	8.151575	-4.535453	-0.138437
H	9.112562	-4.990713	0.083917
C	8.102461	-3.251077	-0.677198
H	9.024077	-2.706455	-0.864905
C	6.844200	1.205393	-1.102483
O	7.448832	1.585052	-2.107708
N	5.831696	1.909056	-0.552527
H	5.307329	1.575724	0.246073
C	5.407997	3.142846	-1.170418
H	5.108360	2.939082	-2.199110
H	6.258837	3.834692	-1.247075
C	4.332148	3.802628	-0.314403
O	4.247468	3.554801	0.887058
N	3.525182	4.713671	-0.923548
C	3.412862	4.940191	-2.371833
H	3.648858	4.035880	-2.933712
H	4.106077	5.729879	-2.693320
C	1.955895	5.367004	-2.551037
H	1.329311	4.474637	-2.631568
H	1.802821	5.979075	-3.443565
C	1.649353	6.110298	-1.247498
H	0.583285	6.178152	-1.048934
H	2.061999	7.124333	-1.272166

C	2.393751	5.283487	-0.175779
H	2.744748	5.917451	0.637972
C	1.517423	4.145507	0.365730
O	1.471117	3.041974	-0.169689
N	0.786632	4.515061	1.442914
H	0.826829	5.490349	1.712875
C	-0.012387	3.600233	2.240341
H	-0.141083	2.691509	1.635145
C	0.671451	3.256930	3.582552
H	-0.021172	2.589232	4.102544
C	1.999012	2.527973	3.354501
H	2.689360	3.120849	2.748463
H	2.482367	2.318936	4.314290
H	1.848938	1.573603	2.846610
C	0.877381	4.502194	4.454467
H	1.299952	4.219517	5.424023
H	1.584646	5.197561	3.987372
H	-0.065092	5.025405	4.636988
C	-1.417209	4.167834	2.446136
O	-2.075401	3.957131	3.467790
N	-1.897252	4.954130	1.457985
H	-1.426007	5.013266	0.560837
C	-3.261455	5.436355	1.521268
H	-3.981775	4.625421	1.362353
H	-3.389492	6.196459	0.750021
C	-4.767002	1.056147	4.138801
H	-5.666016	0.639365	4.577102
C	-4.243443	0.275128	2.927219
H	-4.069913	-0.761579	3.232821
H	-3.271214	0.688290	2.634669
C	-5.141639	0.282047	1.691350
H	-6.084623	-0.245559	1.877579
H	-4.616318	-0.211572	0.868094
S	-5.525886	1.995647	1.136155
C	-6.043958	1.640602	-0.574927
H	-6.895735	0.953899	-0.594980
H	-6.351071	2.587789	-1.023727
H	-5.221354	1.234011	-1.165637
C	-3.702116	1.037911	5.251575
O	-3.562594	0.048728	5.974345
C	0.822263	-1.549258	7.388508
H	1.866805	-1.783453	7.151356

C	0.055660	-1.101532	6.135067	N	-2.011086	-0.044411	-3.890733
H	-0.875739	-0.620047	6.453797	H	-2.188440	0.930616	-4.100184
C	0.873540	-0.093540	5.320155	C	-3.153664	-0.901002	-3.627691
H	1.815117	-0.531182	4.971415	H	-3.009032	-1.831224	-4.181368
H	0.323296	0.243763	4.436414	C	-3.288939	-1.265245	-2.126164
H	1.129815	0.790489	5.913589	H	-3.606662	-0.386635	-1.564289
C	-0.386711	-2.299187	5.266627	H	-2.287327	-1.501125	-1.767524
H	-0.948276	-1.910397	4.407638	C	-4.208645	-2.437527	-1.932904
H	-1.100982	-2.898677	5.844977	C	-5.551918	-2.266073	-1.580237
C	0.740264	-3.207220	4.766386	H	-5.927017	-1.263618	-1.396420
H	0.338373	-4.016543	4.147754	C	-6.398827	-3.366765	-1.448950
H	1.463263	-2.660083	4.155261	H	-7.431584	-3.224340	-1.146392
H	1.285910	-3.667938	5.596794	C	-5.915367	-4.654813	-1.680735
H	-3.903690	7.183628	-2.094146	H	-6.568372	-5.510420	-1.546654
C	-3.233066	6.719088	-2.823182	C	-4.577254	-4.837618	-2.033661
H	-3.820398	6.241239	-3.609154	H	-4.188373	-5.839041	-2.188841
H	-2.628026	7.520418	-3.258518	C	-3.730708	-3.737235	-2.155439
C	-2.318956	5.749410	-2.116180	H	-2.684913	-3.874767	-2.419150
O	-1.602860	6.096975	-1.152573	C	-4.387295	-0.145582	-4.107008
N	-2.342377	4.519181	-2.626974	O	-4.592778	1.018191	-3.740652
H	-3.033819	4.302041	-3.352570	N	-5.245389	-0.789395	-4.925285
C	-1.549637	3.415112	-2.138555	C	-5.077399	-2.117521	-5.498078
H	-0.735639	3.798110	-1.522613	H	-4.985022	-2.877932	-4.718510
C	-2.433776	2.473760	-1.259730	H	-4.205154	-2.168712	-6.157688
H	-3.190753	2.026949	-1.914540	H	-6.049458	-0.238566	-5.193529
H	-2.963572	3.124278	-0.557692	C	-3.073186	-6.206047	0.989698
S	-1.513839	1.196082	-0.346685	H	-2.408912	-7.045279	0.821330
C	-0.990650	2.665026	-3.343368	C	-2.494617	-4.891300	1.111273
O	-1.480705	2.760286	-4.487433	C	-4.409303	-6.369955	1.128801
N	-0.001854	1.803334	-3.051759	H	-4.874046	-7.347988	1.060393
C	0.319927	0.669026	-3.911974	C	-5.325995	-5.240307	1.386258
H	0.228605	0.898311	-4.974532	O	-6.545508	-5.417490	1.493461
C	1.765355	0.198966	-3.666168	C	-4.721714	-3.908623	1.489233
H	1.862688	-0.746718	-4.211603	H	-5.388133	-3.069854	1.650000
C	2.751622	1.215563	-4.253709	C	-3.379682	-3.766489	1.351562
H	2.612726	2.190284	-3.775175	S	-2.470780	-2.262099	1.425977
H	3.785196	0.899855	-4.078344	C	-0.932281	-3.119080	1.226856
H	2.612067	1.346725	-5.332641	C	0.273635	-2.420289	1.296860
C	2.071243	-0.052482	-2.187349	C	0.278482	-0.980785	1.213929
H	1.335964	-0.718850	-1.734829	H	-0.665076	-0.445690	1.210780
H	3.061047	-0.503671	-2.069325	C	1.424948	-0.278952	1.021831
H	2.088239	0.880312	-1.616399	H	1.407328	0.790173	0.839478
C	-0.732310	-0.459887	-3.708502	C	2.710002	-0.958995	0.954562
O	-0.442653	-1.633537	-3.471156	O	3.758575	-0.350302	0.653640

C	2.713794	-2.390026	1.251256
H	3.677196	-2.883479	1.323022
C	1.555388	-3.079873	1.395383
H	1.588907	-4.137057	1.620273
C	-1.162548	-4.525095	1.000707
C	-0.131258	-5.540197	0.582695
O	-0.062317	-6.605723	1.192385
C	0.744435	-5.244524	-0.572618
C	0.563092	-4.144875	-1.423968
H	-0.297588	-3.495476	-1.320461
C	1.494124	-3.836440	-2.409468
H	1.307196	-2.972775	-3.032961
C	1.860812	-6.078978	-0.775587
H	1.984703	-6.936655	-0.123652
C	2.793347	-5.787291	-1.751930
H	3.676301	-6.398625	-1.899946
C	2.631829	-4.641309	-2.552805
O	3.631428	-4.400702	-3.435766
C	3.620043	-3.157706	-4.129667
H	3.604508	-2.316755	-3.428781
H	2.758173	-3.080049	-4.802362
H	4.541852	-3.132021	-4.710924
O	-0.289127	7.461117	1.099801
H	-0.678063	7.302361	0.222898
H	-0.898140	6.969208	1.669092
O	-4.084453	3.669704	-4.741520
H	-4.483617	2.859423	-4.379228
H	-3.181594	3.372230	-4.966397
H	-5.965632	-2.344538	-6.089907
H	-3.454066	5.867328	2.506102
N	-2.928973	2.151875	5.371603
C	-1.804911	2.151184	6.295821
H	-2.897816	2.831446	4.618478
H	-1.244261	3.076686	6.301254
H	-2.165869	1.960374	7.311804
H	-1.105279	1.348733	6.057387
H	0.828345	-0.831853	8.209153
H	0.377678	-2.465309	7.798346
H	-4.985917	2.090525	3.849671
H	6.455014	-0.079262	4.004028
H	5.245949	0.922352	3.161379
H	0.074023	1.606354	-2.040401

MS2

C	-1.679699	7.036070	-3.043572
H	-1.332901	6.423110	-3.878423
H	-2.537208	7.628557	-3.382236
C	-2.075493	6.210174	-1.830117
O	-1.829057	6.562590	-0.671896
N	-2.753042	5.053979	-2.081687
C	-3.245913	4.597832	-3.392405
H	-2.435999	4.547354	-4.124104
H	-4.004029	5.295137	-3.777333
C	-3.841751	3.227328	-3.081420
H	-3.064031	2.463436	-3.076043
H	-4.608918	2.914897	-3.788427
C	-4.382393	3.394520	-1.656706
H	-4.513809	2.434398	-1.160518
H	-5.345775	3.917771	-1.667583
C	-3.334486	4.286666	-0.967518
H	-3.780916	4.988788	-0.256238
C	-2.272597	3.437541	-0.239430
O	-1.797811	2.426861	-0.746892
N	-1.985815	3.870597	1.023222
H	-2.218880	4.844676	1.178037
C	-0.891159	3.362681	1.843129
H	-0.451392	2.472120	1.393894
C	0.238201	4.407343	2.019645
H	0.973627	3.912491	2.659181
C	0.912290	4.750417	0.691325
H	0.217114	5.228553	-0.004292
H	1.741105	5.445417	0.861656
H	1.323346	3.857605	0.219242
C	-0.250017	5.673843	2.735929
H	-0.754993	5.436308	3.677599
H	0.600817	6.321806	2.969324
H	-0.925759	6.262776	2.103764
C	-1.339154	2.836801	3.221269
O	-0.533341	2.658579	4.131026
N	-2.660696	2.550545	3.333174
H	-3.180908	2.594858	2.467837
C	-3.185604	1.702222	4.382012
H	-3.196142	0.647767	4.076086
H	-4.218521	2.003006	4.601153
C	1.458443	3.387547	6.843192
H	2.094205	3.075127	7.678539
C	2.235726	3.498369	5.523016
H	3.128634	4.117779	5.674840
H	1.607236	4.008919	4.789617
C	2.624707	2.143127	4.937498
H	1.718864	1.576207	4.706616
H	3.228946	1.553049	5.637057
S	3.514764	2.236603	3.336574
C	5.055057	3.067317	3.848405
H	5.726087	3.041594	2.986951
H	4.888252	4.108860	4.133668
H	5.526863	2.532047	4.677389
C	9.878376	-1.749153	3.751223
H	9.628981	-1.618671	2.691968
C	8.850099	-1.048711	4.646724
H	8.985730	0.037350	4.560700
H	9.046153	-1.296947	5.699770
C	7.384443	-1.374607	4.315171
H	7.235916	-1.110055	3.258631
C	7.052928	-2.862779	4.484576
H	5.996986	-3.051044	4.265282
H	7.656518	-3.496156	3.826566
H	7.237796	-3.181402	5.518161
C	6.423444	-0.518815	5.148826
H	5.381977	-0.714737	4.877644
H	6.539623	-0.737973	6.217671
H	6.619214	0.548615	5.002702
C	-6.888286	-0.561088	-2.142913
H	-7.301908	-0.990025	-1.227160
C	-5.426536	-0.160035	-2.013779
O	-4.903212	0.589197	-2.836202
N	-4.763174	-0.648971	-0.926190
H	-5.157346	-1.459529	-0.454654
C	-3.303793	-0.584461	-0.858388
H	-2.981166	0.447391	-1.011826
C	-2.888259	-1.062435	0.549319
H	-3.303889	-2.060564	0.674691
H	-3.413010	-0.404992	1.248585
S	-1.111770	-1.117793	1.013161
C	-2.710188	-1.435240	-1.983084
O	-3.375622	-2.223978	-2.651928
N	-1.383741	-1.211144	-2.192727
H	-0.887127	-0.704160	-1.461824
C	-0.663105	-1.965030	-3.190053
H	-1.395706	-2.690914	-3.566581
C	-0.209078	-1.105835	-4.385735

H	0.494089	-1.723785	-4.954743	C	2.189070	2.575527	-2.137996
C	-1.418713	-0.770756	-5.267529	C	0.820450	3.005628	-2.304798
H	-2.186051	-0.247094	-4.687274	H	0.042172	2.543809	-1.709124
H	-1.123980	-0.125400	-6.102533	C	0.473056	3.973768	-3.182045
H	-1.878362	-1.675790	-5.677510	H	-0.561172	4.272207	-3.278365
C	0.486479	0.175326	-3.932975	C	1.459768	4.658713	-4.012997
H	-0.229104	0.837179	-3.440036	O	1.146330	5.566657	-4.799971
H	1.292303	-0.024450	-3.226639	C	2.846069	4.205341	-3.839722
H	0.915997	0.719701	-4.779610	H	3.599829	4.706175	-4.438957
C	0.439447	-2.896312	-2.615486	C	3.184379	3.229025	-2.958295
O	1.268817	-3.411873	-3.382137	H	4.227091	2.965281	-2.838414
N	0.382508	-3.197139	-1.295028	C	3.762791	1.022397	-0.851851
H	-0.284714	-2.700835	-0.688217	C	4.986949	1.032754	-1.734718
C	1.005721	-4.386938	-0.713189	O	5.987195	1.682600	-1.450941
H	1.643723	-4.822340	-1.482785	C	4.907382	0.141440	-2.912100
C	1.856077	-4.040863	0.533929	C	3.833282	-0.739609	-3.087395
H	1.730488	-4.802817	1.307646	H	3.042909	-0.780875	-2.350618
H	1.443341	-3.119911	0.961118	C	3.769379	-1.608418	-4.168766
C	3.336856	-3.881266	0.262230	H	2.924245	-2.282641	-4.240455
C	3.817186	-3.376851	-0.952746	C	5.949846	0.130160	-3.857639
H	3.121918	-3.122839	-1.744213	H	6.785909	0.806073	-3.713103
C	5.187586	-3.228963	-1.171592	C	5.904691	-0.727170	-4.942012
H	5.533591	-2.828529	-2.119071	H	6.696466	-0.748105	-5.683169
C	6.102991	-3.598615	-0.185523	C	4.811546	-1.601853	-5.103233
H	7.169556	-3.488713	-0.359680	O	4.851206	-2.412306	-6.189878
C	5.636440	-4.109225	1.025344	C	3.754745	-3.306112	-6.378255
H	6.336141	-4.396927	1.804562	H	2.814355	-2.759437	-6.508594
C	4.265530	-4.240449	1.247426	H	3.645644	-3.991491	-5.531125
H	3.907793	-4.610749	2.203961	N	-7.330203	-0.647663	2.985833
C	-0.079192	-5.438294	-0.421213	C	-8.591031	-0.941057	2.175977
O	-1.240614	-5.268960	-0.725061	C	-7.595315	0.552743	3.839207
C	0.388759	-6.728030	0.236507	C	-6.944611	-1.799440	3.887718
C	4.830682	-0.455218	0.887466	C	-6.179832	-0.307117	2.062546
H	5.816952	-0.357078	0.448230	H	-8.648999	-0.135237	1.440386
C	3.723975	0.227109	0.268289	H	-9.432613	-0.842479	2.869051
C	4.622006	-1.171698	2.008712	H	-8.405539	0.332549	4.535364
H	5.424587	-1.694808	2.504168	H	-6.684442	0.791037	4.388560
C	3.286146	-1.315228	2.620487	H	-7.869753	1.390664	3.197167
O	3.142778	-1.968986	3.661119	H	-6.894127	-2.731208	3.322245
C	2.152553	-0.674175	1.936765	H	-5.959552	-1.583932	4.302851
H	1.156653	-0.804741	2.347176	H	-7.703341	-1.880700	4.668076
C	2.388182	0.063170	0.823584	H	-5.316244	-0.064455	2.678620
S	1.200283	0.945115	-0.139271	H	-6.485316	0.525547	1.429827
C	2.487235	1.611112	-1.178869	H	-5.937137	-1.188391	1.477537

C	-8.695512	-2.305543	1.494113
H	-9.658027	-2.274282	0.962475
H	-8.748419	-3.114089	2.229421
P	-6.515330	-3.750110	0.972407
O	-7.098144	-4.531758	2.110958
O	-5.218285	-2.981117	1.134018
O	-7.663509	-2.552952	0.575468
O	-6.490486	-4.581253	-0.410018
C	-5.320963	-4.509954	-1.258223
H	-4.413412	-4.760116	-0.705281
H	-5.193037	-3.519389	-1.698324
O	-3.793041	-1.832744	3.404007
H	-2.875554	-1.719499	3.097466
H	-4.201684	-2.361180	2.689152
H	3.982811	-3.868485	-7.284190
H	-7.464110	0.311672	-2.459602
H	-6.964165	-1.314630	-2.926844
H	-0.249067	-7.547456	-0.099508
H	0.279060	-6.639697	1.323156
H	1.438687	-6.948383	0.022990
H	-2.625323	1.766267	5.314815
H	10.880570	-1.335687	3.907766
H	9.939044	-2.819019	3.950862
H	-5.485654	-5.242416	-2.050376
H	-0.861164	7.705845	-2.779861
H	0.639931	2.668862	6.737094
H	1.021878	4.339566	7.062190
